# A showcase of torus canards in neuronal bursters

**DOI:** 10.1186/2190-8567-2-3

**Published:** 2012-02-21

**Authors:** John Burke, Mathieu Desroches, Anna M Barry, Tasso J Kaper, Mark A Kramer

**Affiliations:** 1 Department of Mathematics and Statistics, Center for BioDynamics, Boston University, Boston, MA, 02215, USA; 2 Department of Engineering Mathematics, University of Bristol, Bristol, UK

**Keywords:** Bursting, torus canards, saddle-node of periodic orbits, torus bifurcation, transition to bursting, mixed-mode oscillations, Hindmarsh-Rose model, Morris-Lecar equations, Wilson-Cowan model

## Abstract

Rapid action potential generation - spiking - and alternating intervals of spiking and quiescence - bursting - are two dynamic patterns commonly observed in neuronal activity. In computational models of neuronal systems, the transition from spiking to bursting often exhibits complex bifurcation structure. One type of transition involves the torus canard, which we show arises in a broad array of well-known computational neuronal models with three different classes of bursting dynamics: sub-Hopf/fold cycle bursting, circle/fold cycle bursting, and fold/fold cycle bursting. The essential features that these models share are multiple time scales leading naturally to decomposition into slow and fast systems, a saddle-node of periodic orbits in the fast system, and a torus bifurcation in the full system. We show that the transition from spiking to bursting in each model system is given by an explosion of torus canards. Based on these examples, as well as on emerging theory, we propose that torus canards are a common dynamic phenomenon separating the regimes of spiking and bursting activity.

## 1 Introduction

 The primary unit of brain electrical activity - the neuron - generates a characteristic dynamic behavior: when excited sufficiently, a rapid (on the order of milliseconds) increase then decrease in the neuronal voltage occurs, see for example [1]. This action potential (or ‘spike’) mediates communication between neurons, and therefore is fundamental to understanding brain activity [2-4]. Neurons exhibit many different types of spiking behavior including regular periodic spiking and bursting, which consists of a periodic alternation between intervals of rapid spiking and quiescence, or active and inactive phases, respectively, [5-7]. Bursting activity may serve important roles in neuronal communication, including robust transmission of signals and support for synaptic plasticity [8,9].

 Computational models of spiking and bursting allow a detailed understanding of neuronal activity. Perhaps the most famous computational model in neuroscience - developed by Hodgkin and Huxley [1] - provided new insights into the biophysical mechanisms of spike generation. Subsequently, the dynamical processes that support spiking and bursting have been explored, see for example [10-12]. Recent research has led to a number of classification schemes of bursting, including a scheme by Izhikevich [7] based on the bifurcations that support the onset and termination of the burst’s active phase. This classification requires identifying the separate time scales of the bursting activity: a fast time scale supporting rapid spike generation, and a slow time scale determining the duration of the active and inactive burst phases. This separation of time scales naturally decomposes the full model into a fast system and a slow system. Understanding the bifurcation structure of the isolated fast system is the principal element of the classification scheme. Within this scheme, the onset of the burst’s active phase typically corresponds to a loss of fixed point stability in the fast system, and the termination of the active phase to a loss of limit cycle stability in the fast system. For example, in a fold/fold cycle burster, the former transition occurs through a saddle-node bifurcation (or fold) of attracting and repelling fixed points in the fast system, and the latter transition occurs through a fold of attracting and repelling limit cycles in the fast system. We shall refer to this classification scheme for most of the bursters discussed here.

 Although spiking and bursting have been studied in detail, there are still many interesting questions about the mathematical mechanisms that govern transitions between these states. The spiking state, viewed as a stable periodic orbit of the full system, will lose stability in one of a handful of local bifurcations. However, the transfer of stability to the bursting state involves a wider variety of behavior because it depends more precisely on the global geometry of the system’s phase space. For example, [13] describes a model where the spiking state terminates in a saddle-node bifurcation which simultaneously creates a bursting state (with infinitely long active phase) in the form of an orbit homoclinic to the saddle-node of periodic orbits. The unfolding of this bifurcation - called a blue sky catastrophe - provides a reversible and continuous transition between spiking and bursting dynamics. In contrast, the spiking state in the model studied in [14] can lose stability in either a torus bifurcation or a period doubling bifurcation, depending on secondary parameters. In the latter case, the transition to bursting involves a period doubling cascade to chaos, a feature shared by other models as well [15,16]. The models in [17-19] are further complicated by hysteresis, and include bistable parameter regimes in which both spiking and bursting are stable.

 Recently, it has been proposed that the transition from spiking to bursting can also involve torus canards [20,21]. In this case, the overall transition involves two steps. First, the uniform amplitude spiking state loses stability in a torus bifurcation, leading to amplitude modulated (AM) spiking. Second, the AM spiking state grows into the bursting state by way of a torus canard explosion. The specific torus canard trajectories occur in a small but finite parameter range where the dynamics of the full system move through a fold of limit cycles in the fast system and follow the branch of repelling limit cycles for some time. The torus canard explosion is accompanied by mixed mode oscillations (MMO) which consist of alternating sequences of AM spiking and bursting. The key ingredients for this transition mechanism are a torus bifurcation in the full system and a fold of limit cycles in the fast system, the latter leading to bursting orbits whose active phase terminates in a fold of limit cycles.

In this article, we demonstrate that torus canards arise naturally in computational neuronal models of multiple time scale type. In particular, we show that they arise in well-known neuronal models exhibiting three different classes of bursting: sub-Hopf/fold cycle bursting, circle/fold cycle bursting, and fold/fold cycle bursting. These models are all third order dynamical systems with two fast and one slow variable. We show that these models all have torus bifurcations in the full system, and saddle-node bifurcations of periodic orbits (a.k.a. folds of limit cycles) in the fast systems. In addition, we show that the transitions from spiking to bursting in these systems are given by explosions of torus canards. Based on these observations, we propose that torus canard explosions are a commonly-occurring transition mechanism from spiking to bursting in neuronal models.

 The organization of this manuscript is as follows. In Section 2, we review both the classical canard phenomenon as well as the torus canard phenomenon identified in [21] and recently studied in [20]. In Sections 35, we present the main results describing torus canards at the transition from spiking to bursting in three well-known neuronal models. Finally, we summarize our conclusions in Section 6.

**Remark** Earlier study in [22] examines a two-dimensional map with fast-slow structure in which a fixed point destabilizes into an invariant circle. The small-amplitude oscillations in this map are stable, and the invariant circles exhibit a canard explosion over an interval of parameter values. The map there is piecewise continuous, and conceptually at least could be viewed as a Poincaré map of a higher-order system, with the fixed point representing a periodic orbit and the invariant circle representing a torus, even though in practice the Poincaré maps of smooth systems will be continuous. In addition, we note that in a related two-dimensional map, the transition to chaotic dynamics that occurs when the invariant circles break up has been studied in [23].

**Remark** Throughout this article, we make extensive use of the software package AUTO [24] to carry out the continuation of fixed points and periodic orbits of the models and their fast systems. Bursting trajectories and torus canards are found using direct numerical simulations with a stiff-solver suited to multiple time scale systems, starting from arbitrary initial conditions, and we disregard transients in the figures.

## 2 Overview of canards

In this section, we briefly review the classical phenomenon of canards as they arise in the FitzHugh-Nagumo oscillator, and the recently-identified phenomenon of torus canards as they arise in a Purkinje cell model.

### 2.1 Limit cycle canards

 The FitzHugh-Nagumo (FHN) oscillator [25,26] (or Bonhoeffer-van der Pol oscillator) is a familiar example of a system with planar canards. The system consists of one fast voltage variable *V*, one slow recovery variable *w* and several parameters:

(1a) $$\dot{V}=V-\frac{1}{3}V^{3}-w-I,$$

(1b) $$\dot{w}=\varepsilon \left(V-a-bw\right).$$

To illustrate planar canards, consider system (Equations 1a-1b) with fixed parameters

(2) a=−1.3,b=−0.3,ε=0.05.

Here, *ε* is a small parameter. The *V*-nullcline is a cubic, and it has folds at V=±1. In the limit that ε=0, the full system (Equations 1a-1b) reduces to the fast system in which $\dot{w}=0$ and *w* is a bifurcation parameter for the *V* dynamics. Therefore, for small *ε*, orbits of the full system (Equations 1a-1b) are rapidly attracted to the outer branches (V<−1 and V>1) and repelled away from the middle branch (−1<V<1). Moreover, on long time scales, orbits drift slowly near these branches.

**Fig. 1:**
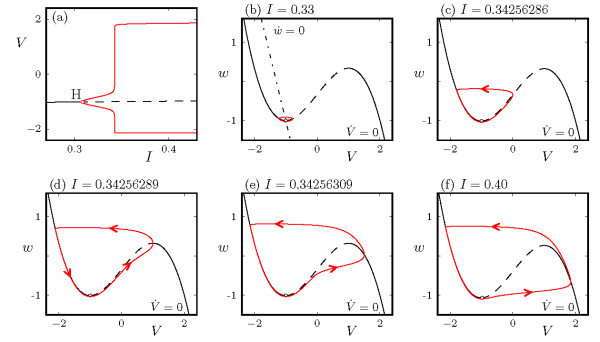
Canards in the FHN system (Equations 1a-1b) at a=−1.3, b=−0.3, ε=0.05. **(a)** Bifurcation diagram of the full system showing fixed points (black curve) and periodic orbits (two red curves, indicating maximal and minimal values of *V* over the orbit). Solid/dashed curves indicate stable/unstable solutions. **(b)**-**(f)** The (V,w) phase plane, including trajectories (red curves) of the full system at several fixed values of *I*. Arrows indicate the direction of flow. Each phase plane also includes the cubic *V*-nullcline $w=V-V^{3}/3-I$, labeled $\dot{V}=0$ and plotted as a solid/dashed curve when it corresponds to a branch of attracting/repelling fixed points of the fast system. The *w*-nullcline w=(V−a)/b, labeled $\dot{w}=0$, is included in (b) but excluded from the other phase space plots for clarity.

The remaining parameter *I* in Equations 1a-1b is the primary control parameter. The behavior of solutions for a sequence of increasing values of *I* is shown in the bifurcation diagram in Figure 1a. At small values of *I*, the system exhibits an attracting fixed point. As *I* increases, the fixed point loses stability in a supercritical Hopf bifurcation (H, at I≃0.3085) and a stable limit cycle appears. The limit cycle is initially of small amplitude, growing as the square root of the distance from onset, but rapidly increases in amplitude over a small range of *I* near I=0.34256289. Frames (b)-(f) of Figure 1 show the growth of the periodic orbit in the (V,w) phase plane. The linear *w*-nullcline and cubic *V*-nullcline are included for later reference. 

This rapid transition from small to large amplitude oscillations is known as a canard explosion, and it is readily understood using phase plane analysis, aided by a fast-slow decomposition. The fixed point of the full system (Equations 1a-1b), which occurs at the intersection of the *V*- and *w*-nullclines, is stable for small values of *I* where the intersection occurs in V<−1 (i.e., on the segment of the *V*-nullcline which corresponds to attracting fixed points of the fast system) and unstable at larger *I* where the intersection occurs in −1<V<1 (i.e., on the segment of the *V*-nullcline which corresponds to repelling fixed points of the fast system). The Hopf bifurcation at I≃0.3085 occurs as the intersection of the nullclines moves through the fold of fixed points of the fast system at V=−1, or more precisely when the intersection is at $V^{2}=1-b\varepsilon$. The small amplitude oscillations that occur for nearby values of *I* are confined to a relatively small region in phase space surrounding the fold of fixed points of the fast system (see Figure 1b for a sample orbit at I=0.33).

At I∼0.3425 the periodic orbits rapidly increase in amplitude in a canard explosion. The first canard orbits, referred to as ‘headless ducks’ (trajectory in Figure 1c), correspond to periodic orbits of the full system that spend O(1) time in the neighborhood of two of the three branches of fixed points of the fast system: the trajectory drifts toward smaller *w* along the left attracting branch, and drifts toward larger *w* along the repelling middle branch before returning back to the attracting branch. With further increase of the parameter *I*, the canard orbit grows in amplitude and moves further along the repelling branch, eventually reaching the second fold of fixed points of the fast system. This corresponds to the maximal canard (Figure 1d). Beyond this value of *I*, the canard orbits spend O(1) time in the neighborhood of all three branches of fixed points of the fast system, forming canard trajectories referred to as ‘ducks with heads’ (trajectory in Figure 1e). As the parameter *I* increases further, the trajectory leaves the repelling branch sooner, eventually resulting in relaxation oscillations (Figure 1f), in which the trajectory spends O(1) time near both branches of attracting fixed points of the fast system.

 Just as is the case for canards in the van der Pol equation, a formula is known for the critical parameter value, $I_{c}(\varepsilon )$, at which the maximum headless canard exists in the FitzHugh-Nagumo equations. This critical value is the unique one for which the attracting and repelling slow manifolds coincide, and it is given, for example, in [27]. Moreover, from the theory of limit cycle canards, it is known that the entire canard explosion takes place in a parameter interval of exponentially small width in *ε* about this critical value.

 The common feature among the canard trajectories is that they periodically spend O(1) time drifting along the branch of repelling fixed points of the fast system. The crucial distinction between the canards with and without heads is the direction in which they leave the repelling branch. We note that for the parameter values chosen in Equations 1a-1b, the Hopf bifurcation is supercritical. Other parameter choices can make this Hopf bifurcation subcritical, resulting in bistablility between the fixed point and relaxation oscillation. In that case the small amplitude oscillations near onset and the headless canards are unstable, the maximal canard corresponds to a saddle-node of periodic orbits of the full system, and the canards with heads and the relaxation oscillations are stable. In addition, the canards with and without heads coexist in phase space at the same *I* values. A more detailed description of the classical phenomenon of canards in planar systems and analysis techniques can be found in [28-31].

### 2.2 Torus canards

 In the classical canards described above, the dynamics of the full system undergo a Hopf bifurcation and, after passing through a fold of fixed points in the fast system, canard trajectories follow a branch of repelling fixed points for some time. We regard the torus canard as the one-dimension-higher analog of this classical canard because the fundamental components of a torus canard are of one dimension higher than the corresponding components in a limit cycle canard. For systems with a torus canard, there are families of attracting and repelling limit cycles of the fast system that meet in a saddle-node, whereas for systems with a limit cycle canard these are families of equilibria. As a result, a torus canard is a quasi-periodic orbit, whereas the planar canards described above are periodic. Also, for systems with a torus canard, there is a torus bifurcation in the full system, whereas systems with limit cycle canards include a Hopf bifurcation. We now review the essential features of the torus canards in a Purkinje cell model [21]. This single-compartment model consists of five ordinary differential equations that describe the dynamics of the membrane potential, *V*, and four ionic gating variables, , and : (3a)(3b)(3c)(3d)(3e)

 The ionic gating variables represent: a leak current ( term), a high-threshold noninactivating calcium current ( term), a transient inactivating sodium current ( term), a delayed rectifier potassium current ( term), and a muscarinic receptor suppressed potassium current or M-current ( term). The forward and backward rate functions ($\alpha_{X}$ and $\beta_{X}$ for X=CaH,NaF,KDR,KM) and fixed parameter values are given in Appendix 1. The parameter *J* represents an externally applied current, and is the primary control parameter considered here.

**Fig. 2:**
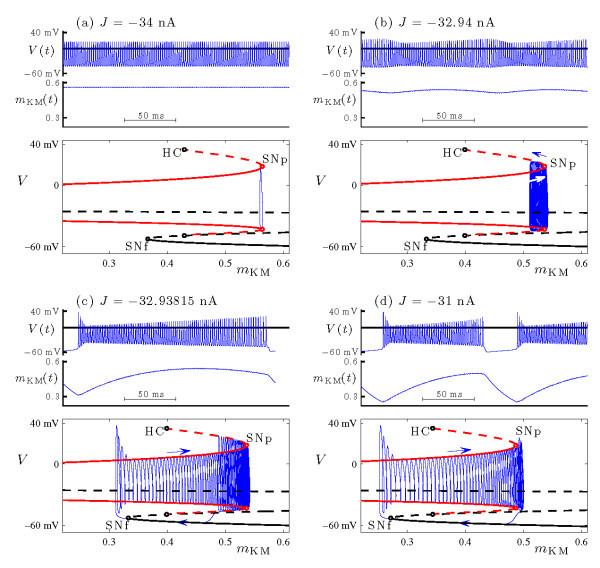
Dynamics of the Purkinje cell model (Equations 3a-3e) at several values of *J*: **(a)** rapid spiking, at J=−34 nA; **(b)** AM spiking or headless torus canard, at J=−32.94 nA; **(c)** torus canard with head, at J=−32.93815 nA; **(d)** standard fold/fold cycle bursting at J=−31 nA. The upper and middle panel of each frame show, respectively, the time series of the voltage *V* and the slow gating variable . In the lower panel of each frame, the trajectory of the full system (blue curve) is plotted in projection in the  phase space, along with the bifurcation diagram of the fast system at the corresponding value of *J*. The bifurcation diagrams include branches of fixed points (black curves) and periodic orbits (two red curves, indicating maximal and minimal values of *V* over the orbit). Solid/dashed curves indicate stable/unstable solutions of the fast system. The labels mark saddle-node bifurcations of fixed points (SNf), saddle-node bifurcations of periodic orbits (SNp), and homoclinic bifurcations (HC). Arrows indicate the direction of drift in  for the trajectories.

Figure 2 illustrates the transition from spiking to bursting in the Purkinje cell model (Equations 3a-3e) as *J* increases. We begin with a description of the voltage dynamics, shown in the upper panel of each frame for fixed *J*, with *J* increasing from frames (a) to (d). For *J* sufficiently negative, the system exhibits a stable periodic orbit which corresponds biophysically to a uniform amplitude spiking state. At J≃−32.96 nA this periodic orbit undergoes a supercritical torus bifurcation and stability transfers to orbits on the five-dimensional phase space torus. The voltage trace of such an orbit exhibits AM spiking. Very close to the torus bifurcation, the modulation is weak (not shown in the figure) but near J=−32.94 nA the amplitude modulation increases significantly (Figure 2b). Further increase of *J* causes the system to transition from AM spiking to bursting (Figure 2c, at J=−32.93815 nA), which persists upon additional increases in *J* (Figure 2d, at J=−31 nA). In the transition region between J=−32.94 nA and J=−32.93815 nA, MMO appear which consist of alternating sequences of AM spiking and bursting orbits (not shown). 

As was the case in the FHN model, the behavior of the Purkinje model can be understood by decomposing Equations 3a-3e into fast and slow systems. The separation of time scales is apparent in Figure 2, which also includes time-series plots of the M-current gating variable  for each fixed *J* (middle panel of each frame). This gating variable evolves on a much slower time scale than the other four variables - typically by about a factor of ten. Hence, the dynamics of system (Equations 3a-3e) may be studied by focusing on the bifurcation structure of the four-dimensional fast system which is defined by setting  and treating  as a bifurcation parameter. Figure 2 includes bifurcation diagrams of this fast system for each fixed *J* (lower panel of each frame). In each case, the bifurcation diagram of the fast system has the same qualitative features, including an S-shaped branch of fixed points and a branch of periodic orbits. The latter are stable at small values of , lose stability in a saddle-node bifurcation (SNp) and terminate in a homoclinic bifurcation (HC). The slow drift of solutions of the full system is determined by the  equation in Equations 3a-3e. Each frame in Figure 2 includes the trajectory of the full system plotted in projection on the  phase space - i.e., superimposed on the bifurcation diagram of the fast system.

 In Figure 2a, the spiking orbit of the full system remains near the branch of attracting periodic orbits of the fast system and does not drift in  because  when averaged over the fast period (see [19] for a description of the averaging procedure). The torus bifurcation of the full system occurs when the rapid spiking state lies close to the saddle-node of periodic orbits of the fast system, and the weakly modulated spiking states lie on the phase space torus which surrounds this saddle-node. The first torus canard orbits emerge at slightly larger values of *J* as the torus rapidly increases in amplitude. The AM spiking state in Figure 2b corresponds to a headless torus canard which spends long times (i.e., many fast oscillations) near branches of both attracting and repelling periodic orbits of the fast system. The trajectory drifts along the former in the direction of increasing  (toward SNp) and along the latter in the direction of decreasing  (away from SNp). When it leaves the repelling branch, it returns directly to the branch of attracting orbits and repeats the cycle. The long-period bursting state in Figure 2c corresponds to a torus canard with head which spends long times near branches of both attracting and repelling periodic orbits of the fast system, but leaves the repelling branch for the branch of attracting fixed points of the fast system, corresponding to the onset of the inactive phase of the burst. During the inactive burst phase the trajectory drifts in the direction of decreasing , and eventually reaches the saddle-node of fixed points of the fast system (SNf). It then transitions back to the branch of attracting periodic orbits of the fast system to begin the active phase of the burst.

 At the larger value of *J* in Figure 2d, the bursting trajectory no longer corresponds to a canard because it does not spend any time along the branch of repelling periodic orbits of the fast system. Instead, the trajectory corresponds to a standard fold/fold cycle burster [7] in which the active phase of the burst begins at a saddle-node of fixed points (SNf) and ends at a saddle-node of periodic orbits (SNp). The transition from AM spiking to bursting corresponds to the torus canard explosion from headless ducks to ducks with heads. The MMO that occur during the transition are an expected consequence of the theory of torus canards [20].

 Torus canard-like trajectories have been observed in other models of neuronal dynamics. They were described in [32] in the context of an abstract model consisting of a planar fast-slow system that is rotated about an axis. Similar dynamics in systems without rotational symmetry were described in [22,23] using a map-based model in two dimensions, and in [20] by examining the intersections of invariant manifolds in a continuous-time model in three dimensions. The abstract model of [20] shares the same key ingredients as the Purkinje cell model of [21] described above: a torus bifurcation in the full system and a fold of limit cycles in the fast system. Moreover, the torus canards in that model also undergo an explosion involving headless ducks, MMO, and ducks with heads, and they occur in the transition regime between spiking and bursting. In the following three sections, we show that torus canards also occur in three neuronal models with different classes of bursting dynamics.

## 3 Torus canards in the Hindmarsh-Rose system

 We begin with the following modified version of the Hindmarsh-Rose (HR) system [33] developed in [34]

(4a) $$\dot{x}=sax^{3}-sx^{2}-y-bz,$$

(4b) $$\dot{y}=\phi (x^{2}-y),$$

(4c) $$\dot{z}=\varepsilon (sa_{1}x+b_{1}-kz).$$

The small parameter ε≪1 induces a separation of time scales, so that the voltage variable *x* and the gating variable *y* are fast and the recovery variable *z* is slow.

 The HR model is known to exhibit rich dynamics, including square-wave bursting (a.k.a. plateau bursting) and pseudo-plateau bursting [34]. Here, we show that this model also exhibits sub-Hopf/fold cycle bursting (in which the active phase of the burst initiates in a subcritical Hopf bifurcation and terminates in a fold of limit cycles), and that torus canards occur precisely in the transition region from spiking to this type of bursting. To do so, we first describe the behavior of the HR system (Equations 4a-4c) as it transitions from spiking to bursting dynamics, and show that this occurs near a torus bifurcation of the full system (Section 3.1). We then analyze the fast system of the HR model, and show that it includes a saddle-node of periodic orbits (Section 3.2). Once these key ingredients are identified, we show (Section 3.3) that the full HR model includes a torus canard explosion, and that it lies in the transition region between spiking and bursting.

As we carry out this dynamical systems analysis, we will show how the voltage dynamics change as the system parameters are varied through the transition regime between spiking and bursting. We will show that, during spiking, the voltage variable *x* exhibits the characteristic, but idealized, features of regular, periodic oscillations. By contrast, during bursting, the voltage traces exhibit, in alternation, an active phase of rapid spiking (with slowly changing spiking amplitude) and a quiescent phase during which the voltage stays near a stable equilibrium level.

In the transition regime between spiking and bursting, the voltage traces associated to the torus canards gradually morph between these two types of behavior. In particular, we will show that the headless torus canards correspond to amplitude modulation in the voltage; the maximal torus canard corresponds to the voltage trace for which bursting first arises; and, the torus canards with heads have voltage traces associated to them that are similar to those seen in the bursting regime. In this manner, the transition between spiking and bursting happens smoothly for the voltage traces, and there are some well-defined transition points along the way. As a caveat, we note that, while the headless torus canards correspond to amplitude-modulation, not all AM solutions in neuronal models are torus canards.

 We treat $b_{1}$ as the primary control parameter, meaning that we examine the transition from spiking to bursting as $b_{1}$ varies. Because $b_{1}$ only occurs in Equations 4a-4c in the slow $\dot{z}$ equation, the bifurcation diagram of the fast system of Equations 4a-4c will be identical for all $b_{1}$. We anticipate that different trajectories exhibited by the system at different $b_{1}$ follow different paths around this same bifurcation diagram, as determined by the slow equation. We take *s* as a secondary control parameter, and examine how the transition from spiking to bursting behaves at different values of *s*. Of particular interest are the changes in the dynamics of the full system that can be explained by changes in the bifurcation structure (such as codimension-2 bifurcations) of the fast system. Except where otherwise noted, we set the remaining parameters to

(5) $$a=0.5,\;\phi =1,\;a_{1}=-0.1,\;k=0.2,\;b=10,\;\varepsilon =10^{-5},$$

which are based on the values used in [34].

### 3.1 Dynamics of the full system

**Fig. 3:**
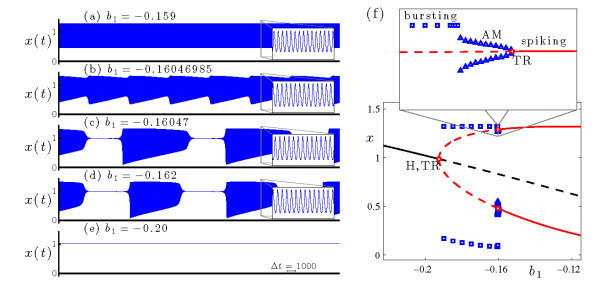
Dynamics of the HR system (Equations 4a-4c) at s=−1.95. **(a)-(e)** Time series of the voltage variable *x* at several values of $b_{1}$, with $b_{1}$ decreasing from top to bottom. **(f)** Bifurcation diagram, including branches of fixed points (black curve) and periodic orbits (two red curves, indicating maximal and minimal values of *x* over the orbit). Solid/dashed curves indicate stable/unstable solutions. The Hopf bifurcation (H) at $b_{1}≃-0.1927$ is supercritical. The torus bifurcations (TR) at $b_{1}≃-0.1926$ and $b_{1}≃-0.1603$ are also supercritical. Direct numerical simulations show that the system exhibits bursting (□, indicating maximal and minimal values of *x*) and AM spiking (△, indicating extrema of the modulation envelope) at values of $b_{1}$ between the two torus bifurcations.

 Figure 3 shows the dynamics of the HR model (Equations 4a-4c) at s=−1.95. The time series of the voltage variable *x* illustrate the transition of interest: at $b_{1}=-0.159$ the system exhibits uniform amplitude rapid spiking (Figure 3a), and at $b_{1}=-0.162$ the system exhibits bursting (Figure 3d). In what follows, we show that this transition involves torus canards (examples of which are shown in frames (b) and (c) of Figure 3). Further decrease of $b_{1}$ eventually leads to fixed point dynamics (Figure 3e). The transition from bursting to quiescence is a separate topic and is beyond the scope of this article, but similar transitions have been studied in other models [10,14,15]. 

The first key ingredient for the emergence of torus canards is the presence of a torus bifurcation in the full system, at the boundary of the regime of rapid spiking. To see that this occurs in the HR system (Equations 4a-4c), consider the bifurcation diagram of the full system shown in Figure 3f. The rapid spiking state in Figure 3a lies on a branch of attracting periodic orbits which loses stability in a supercritical torus bifurcation (TR) at $b_{1}≃-0.1603$. The bursting dynamics shown in Figure 3d occur at more negative values of $b_{1}$, where the periodic orbits remain unstable. For completeness we note that the unstable branch of periodic orbits regains stability in another torus bifurcation (TR) at $b_{1}≃-0.1926$ almost immediately before coalescing with the branch of fixed points in a supercritical Hopf (H) bifurcation at $b_{1}≃-0.1927$.

### 3.2 Bifurcation analysis of the fast system

**Fig. 4:**
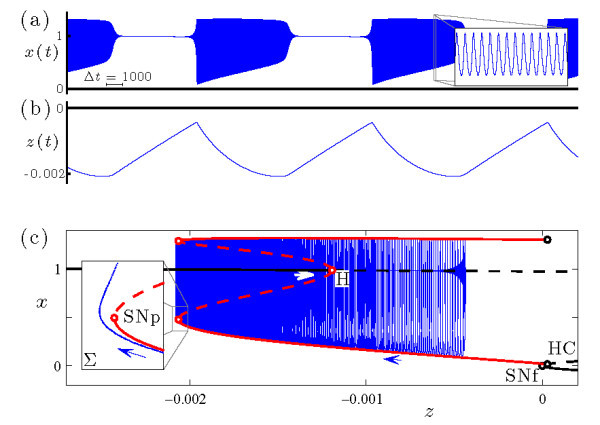
An example of sub-Hopf/fold cycle bursting in the HR system (Equations 4a-4c), with $\left(b_{1},s)=(-0.162,-1.95\right)$. The other parameter values are given by Equation 5. **(a)** Time series of the fast *x* variable. **(b)** Time series of the slow *z* variable. **(c)** The bursting trajectory (blue curve) plotted in projection onto the (z,x) phase space, along with the bifurcation diagram of the fast system at this value of *s*. The bifurcation diagram includes branches of fixed points and periodic orbits, and follows the plotting conventions in Figure 2. The inset shows the Poincaré map of the bursting trajectory near SNp, also plotted in projection onto the (z,x) phase space. The Poincaré surface $\Sigma \equiv \{(x,y,z)|0=sax^{3}-sx^{2}-y-bz\}$ is chosen so that the iterates correspond to local extrema in *x* of the trajectory.

Figure 4 shows in more detail the bursting dynamics exhibited by system (Equations 4a-4c) at s=−1.95 and $b_{1}=-0.162$, previously included in Figure 3d. Inspection of the *x* time series (Figure 4a) reveals the two phases of the burst, consisting of periodic intervals of spiking and quiescence. The variable *z* (Figure 4b) slowly decreases during the active phase of the burst and slowly increases during the inactive phase. Figure 4c shows the bursting trajectory plotted in projection onto the (z,x) phase space. 

 To explore the dynamical mechanism responsible for the bursting state, it is convenient to consider the fast-slow decomposition of this system. The fast system of Equations 4a-4c is obtained by setting ε=0 and treating the slow variable *z* as a bifurcation parameter. The classification of the dynamics in Figure 4 as a sub-Hopf/fold cycle burster is understood by examining the trajectory of the full system in relation to the bifurcation diagram of the fast system. During the quiescent phase of the burst, the trajectory of the full system increases in *z* along the branch of fixed points of the fast system. The active phase of the burst initiates when the trajectory passes through the subcritical Hopf bifurcation (H, at z≃−0.0012) and, after a slow passage effect [35,36] (which causes the orbit to stay near the branch of repelling fixed points for some time), spirals out to the attracting branch of periodic orbits. During the active phase of the burst, the trajectory of the full system shadows the attracting branch of periodic orbits of the fast system as it drifts to smaller *z* values. The active phase terminates when the trajectory falls off the branch of periodic orbits at a saddle-node bifurcation (SNp, at z≃−0.0021) and spirals back in toward the attracting branch of fixed points of the fast system to repeat the cycle. Note that the bifurcation diagram reveals a key ingredient required for torus canards: a saddle-node of periodic orbits in the fast system.

### 3.3 Torus canard explosion

The transition from spiking to bursting as $b_{1}$ decreases through the torus bifurcation at $b_{1}≃-0.1603$ occurs by way of a torus canard explosion. When $b_{1}$ exceeds the torus bifurcation value, the periodic orbit of the full system is stable (Figure 3a). This trajectory resembles a periodic orbit taken from the attracting branch of periodic orbits of the fast system, and does not drift in *z* because $\dot{z}=0$ for this orbit when averaged over the fast period. The torus bifurcation at $b_{1}≃-0.1603$ creates a phase space torus that surrounds the saddle-node of periodic orbits of the fast system. Near onset, this leads to weak amplitude modulation of the spiking state as the trajectory winds around the phase space torus. Further decrease of $b_{1}$ causes the amplitude modulation to increase as the phase space torus grows. The bifurcation diagram in Figure 3 clearly shows a pronounced increase in the amplitude modulation near $b_{1}=-0.16046$. This occurs as the trajectory shadows, in alternation, parts of the attracting and repelling branches of periodic orbits of the fast system. As $b_{1}$ decreases, this leads first to headless torus canards, then torus canards with heads, and finally sub-Hopf/fold cycle bursting.

**Fig. 5:**
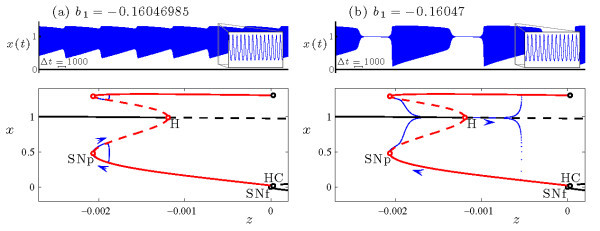
Torus canards in the HR system (Equations 4a-4c) at s=−1.95: **(a)** torus canard without head at $b_{1}=-0.16046985$, and **(b)** torus canard with head at $b_{1}=-0.16047$. In each frame, the upper panel shows the time series of the *x* variable (previously included in Figure 3), and the lower panel shows the Poincaré map of the torus canard trajectory in phase space. The bifurcation diagrams of the fast system include branches of fixed points and periodic orbits, and follow the plotting conventions in Figure 2.

The time series of the voltage variable of a headless torus canard resembles AM spiking, and that of a torus canard with head resembles bursting. The classification of a trajectory as a torus canard is only made clear by examining it in phase space. To illustrate the distinction in more detail, Figure 5 shows two sample trajectories from the torus canard explosion sequence. Both types of torus canards spiral on the fast time scale, following the envelope of the outer (attracting) branch of periodic orbits of the fast system to the fold (SNp) and then continuing for some time along the envelope of the inner (repelling) branch of periodic orbits. The trajectory shown in Figure 5a at $b_{1}=-0.16046985$ leaves the branch of repelling periodic orbits and returns directly to the attracting branch of periodic orbits, forming a headless torus canard. As $b_{1}$ is decreased, the length of time that the headless torus canard orbit spends near the branch of repelling periodic orbits increases. Further decrease in $b_{1}$ results in a narrow region of MMO behavior (not shown) followed by torus canards with heads, as shown in Figure 5b at $b_{1}=-0.16047$. Now, the trajectory leaves the branch of repelling periodic orbits for the branch of attracting fixed points. The trajectory then drifts to larger *z*, leaves the branch of fixed points after a slow passage through the Hopf bifurcation, returns to the branch of attracting periodic orbits, and the cycle repeats. 

This bifurcation sequence, consisting of a family of headless torus canards (Figure 5a) followed by MMO and a family of torus canards with heads (Figure 5b), constitutes a torus canard explosion. The torus canard explosion marks the transition from AM spiking to bursting, which is the final stage in the overall transition from spiking to bursting in this model. When $b_{1}$ is sufficiently negative (i.e., sufficiently past the torus canard explosion), the trajectory does not follow the branch of repelling periodic orbits and instead transitions directly from the saddle-node of periodic orbits to the branch of attracting fixed points, resulting in a large amplitude sub-Hopf/fold cycle bursting orbit such as the one shown in Figure 4 at $b_{1}=-0.162$.

### 3.4 Two-parameter bifurcation diagram and relation to other types of bursting

**Fig. 6:**
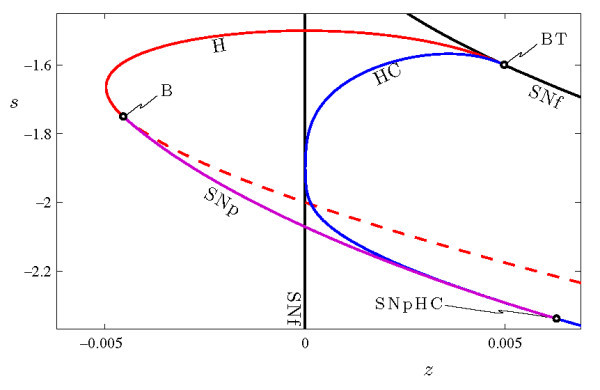
Two-parameter bifurcation diagram of the fast system of Equations 4a-4c in the (z,s)-plane. The loci of Hopf H (red curve) and homoclinic HC (blue curve) bifurcations emerge from the Bogdanov-Takens point BT at (z,s)≃(0.004985,−1.599). The curve H is plotted as a solid/dashed line when the Hopf bifurcation is supercritical/subcritical. The saddle-node of periodic orbits SNp (purple curve) exists between the Bautin bifurcation point B at (z,s)≃(−0.004541,−1.75) and the SNpHC at (z,s)≃(0.006291,−2.339).

To characterize the range over which torus canards may occur, we consider how the bifurcation structure of the fast system changes with the parameter *s*. To this end, we compute loci of the codimension-1 bifurcations from Figure 4 in the (z,s) parameter plane of the fast system. The results are shown in Figure 6. There are three noteworthy codimension-2 bifurcation points included in this figure. The loci of Hopf (H) and homoclinic (HC) bifurcations emerge from the saddle-node of fixed points at a Bogdanov-Takens point (BT). A Bautin bifurcation (B) marks the point at which the Hopf bifurcation changes from supercritical to subcritical, and also the associated emergence of the curve of saddle-node of periodic orbits (SNp). Finally, this SNp curve ends when it collides with the homoclinic bifurcation at the point labeled SNpHC. This final codimension-2 bifurcation amounts to a change in the criticality of the homoclinic bifurcation. Thus, the HR model’s fast system includes a saddle-node of periodic orbits for values of *s* within the range −2.3388≤s≤−1.75. The torus bifurcation in the full system persists over this range as well, so we expect the system will also include torus canards over a range of *s* values. 

**Fig. 7:**
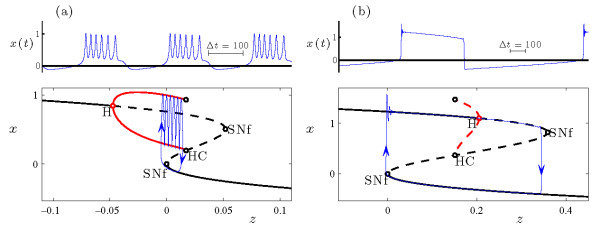
Examples of different bursting types in the HR system (Equations 4a-4c): **(a)** square-wave bursting for s=−1.61, and **(b)** pseudo-plateau bursting for s=−2.6. Other parameters are as in Equation 5, except b=1, ε=0.004, and $b_{1}=-0.03$. Plotting conventions follow Figure 2.

 The HR system (Equations 4a-4c) also exhibits a wide range of different bursting behavior beyond the sub-Hopf/fold cycle bursting described above. Some of this behavior can be understood by examining Figure 6. For example, increasing *s* eliminates SNp by changing the Hopf bifurcation from subcritical to supercritical. This can lead to the square-wave bursting shown in Figure 7a. There, the active phase of the burst is initiated at a saddle-node of fixed points SNf and terminates at a homoclinic bifurcation HC. The classification of this burster is now fold/homoclinic, and an essential ingredient for torus canards - a saddle-node of periodic orbits in the fast system - is lost. Therefore, the torus canard phenomenon is also lost. This type of burster has been studied in [34,37-39]. 

 Decreasing the parameter *s* also eliminates the saddle-node of periodic orbits. In this case, the Hopf bifurcation H remains subcritical and the saddle-node of periodic orbits SNp is eliminated when it collides with the homoclinic bifurcation HC. This can lead to pseudo-plateau bursting, as shown in Figure 7b, which has been studied extensively in [34,39]. In this case, the active phase of the burst again initiates at the saddle-node of fixed points SNf, but these oscillations (which are associated with the complex eigenvalues of the upper fixed point, not the periodic orbits) terminate after the slow passage through the subcritical Hopf bifurcation. Here again, the elimination of an essential ingredient - the saddle-node of periodic orbits - results in the loss of the torus canard phenomenon.

In conclusion, the HR system exhibits different types of bursting behavior depending on the choice of parameter *s*. For a wide range of *s*, sub-Hopf/fold cycle bursting occurs. We have shown that, for this type of bursting, a torus bifurcation occurs between the regimes of rapid spiking and bursting, and that a torus canard explosion separates the two.

## 4 Torus canards in the Morris-Lecar-Terman system

 In this section we consider a version of the Morris-Lecar system [37] extended to $R^{3}$ by Terman [15], which we call the Morris-Lecar-Terman (MLT) model. The equations are

(6a) $$\dot{V}=y-g_{L}(V-E_{L})-g_{K}w(V-E_{K})-g_{\mathrm{Ca}}m_{\infty }(V)(V-E_{\mathrm{Ca}}),$$

(6b) $$\dot{w}=-\frac{w-w_{\infty }(V)}{\tau_{w}(V)},$$

(6c) $$\dot{y}=\varepsilon (k-V),$$

where

(7a) $$m_{\infty }(V)=\frac{1}{2}\left[1+\tanh\left(\frac{V-c_{1}}{c_{2}}\right)\right],$$

(7b) $$w_{\infty }(V)=\frac{1}{2}\left[1+\tanh\left(\frac{V-c_{3}}{c_{4}}\right)\right],$$

(7c) $$\tau_{w}(V)=\tau_{0}\mathrm{sech}\left(\frac{V-c_{3}}{2c_{4}}\right).$$

The MLT model exhibits a wide variety of bursting dynamics. It was examined by Terman [15] in a parameter regime in which it exhibits fold/homoclinic bursting. In addition, the same model was used in [7] to illustrate both circle/fold cycle bursting and fold/homoclinic bursting. Here, we focus on system (Equations 6a-6c) as an example of circle/fold cycle bursting, in which the active phase of the burst initiates in a saddle-node bifurcation on an invariant circle (i.e., SNIC) and terminates in a fold of limit cycles. We find torus canards in this model, precisely in the transition regime from spiking to this type of bursting.

This section follows the same outline used in the previous section. First, we show that the spiking state in the full MLT model loses stability in a torus bifurcation (Section 4.1), and that this occurs near a fold of limit cycles in the fast system (Section 4.2). Once these key ingredients are identified, we show that this system includes a torus canard explosion in the transition regime between spiking and bursting (Section 4.3).

 In what follows, we treat *k* and $g_{\mathrm{Ca}}$ as the primary and secondary control parameters, respectively. The remaining system parameters are fixed at

(8a) $$g_{L}=0.5,\;g_{K}=2,\;E_{L}=-0.5,\;E_{K}=-0.7,\;E_{\mathrm{Ca}}=1,$$

(8b) $$\begin{array}{rl} c_{1} & =-0.01,\;c_{2}=0.15,\;c_{3}=0.1,\\ c_{4} & =0.16,\;\tau_{0}=3,\;\varepsilon =0.003, \end{array}$$

which are the values used in [7].

### 4.1 Dynamics of the full system, and a torus bifurcation

**Fig. 8:**
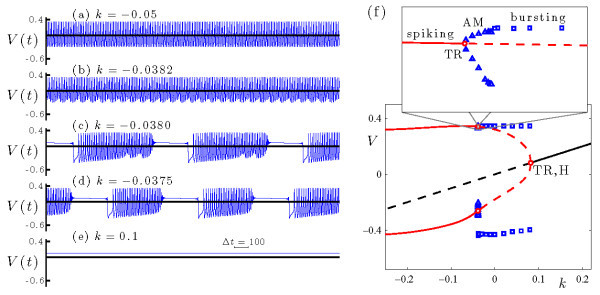
Dynamics of the MLT system (Equations 6a-6c) at $g_{\mathrm{Ca}}=1.25$. **(a)-(e)** Time series of the voltage variable *x* at several values of *k*, with *k* increasing from top to bottom. **(f)** Bifurcation diagram, including branches of fixed points (black curve) and periodic orbits (two red curves, indicating maximal and minimal values of *V* over the orbit). Solid/dashed curves indicate stable/unstable solutions. The torus bifurcations at k≃−0.03852 and k≃0.08184 are both supercritical. Direct numerical simulations show that the system exhibits bursting (□, indicating maximal and minimal values of *V*) and AM spiking (△, indicating extrema of the modulation envelope) at values of *k* between the two torus bifurcations.

The dynamics of the full MLT model (Equations 6a-6c) at $g_{\mathrm{Ca}}=1.25$ is shown in Figure 8. The time series of the voltage variable *V* reveals the transition from spiking (Figure 8a) to bursting (Figure 8d) as the primary bifurcation parameter *k* increases. The transition region is dominated by AM spiking (Figure 8b). 

The bifurcation diagram in Figure 8f shows that the uniform amplitude spiking state lies on a branch of attracting periodic orbits which are stable for sufficiently negative values of *k*. As *k* increases, the periodic orbits lose stability in a supercritical torus bifurcation at k≃−0.03852. Beyond this torus bifurcation value, bursting appears in the full system followed by a restabilization of periodic orbits in a second torus bifurcation. Finally, for slightly larger *k*, just beyond this second torus bifurcation, there is a Hopf bifurcation. The periodic orbits disappear in this Hopf bifurcation, and the fixed points become stable. This highly depolarized (i.e., large *V*) fixed point corresponds to the physiological state of depolarization block in the MLT system (Figure 8e). Again, the transition from bursting to quiescence is beyond the scope of this article.

The transition from spiking to bursting shown in Figure 8 for the MLT model is clearly reminiscent of the same transition shown in Figure 3 for the HR model. The different direction for this transition (from right to left as $b_{1}$ decreases in Figure 3, and from left to right as *k* increases in Figure 8) is a trivial consequence of sign conventions in defining the equations of motion for the two systems. The different separations between fast and slow time scales (so that each burst in Figure 3 includes several thousand spikes while those in Figure 8 include only a few dozen) is a consequence of the different values of *ε* in the different models. A more important distinction is the two different classes of bursting represented: sub-Hopf/fold cycle bursting in Figure 3, and circle/fold cycle bursting in Figure 8.

### 4.2 Bifurcation analysis of the fast system

**Fig. 9:**
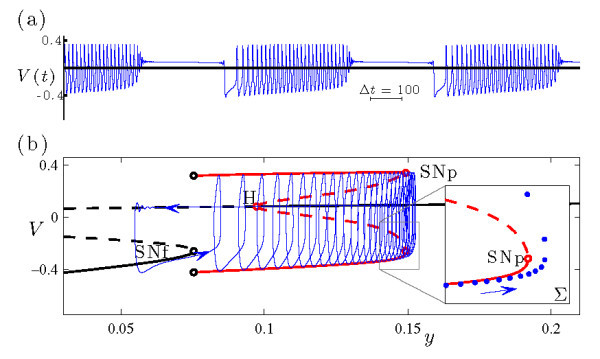
An example of circle/fold cycle bursting in the MLT system (Equations 6a-6c) at $\left(k,g_{\mathrm{Ca}})=(-0.0375,1.25\right)$. **(a)** Time series of the fast *V* variable. **(b)** The bursting trajectory (blue curve) is plotted in projection onto the (y,V) phase space along with the bifurcation diagram of the fast system at this value of $g_{\mathrm{Ca}}$. Plotting conventions follow Figure 2. The inset shows the Poincaré map of the bursting trajectory near SNp, also plotted in projection onto the (y,V) phase space. The Poincaré surface Σ is chosen so that the iterates correspond to local extrema in *V* of the trajectory.

Figure 9 shows the circle/fold cycle bursting orbit from system (Equations 6a-6c) at k=−0.0375 and $g_{\mathrm{Ca}}=1.25$. The upper frame shows the *V* time series, and the lower frame plots the bursting trajectory in projection into the (y,V) phase space. 

 The 2D fast system of Equations 6a-6c, obtained by setting ε=0 and treating *z* as a bifurcation parameter, is the familiar Morris-Lecar system [37]. In Figure 9b, it is clear that the active phase of the burst ends when the trajectory falls off the branch of attracting periodic orbits of the fast system at a saddle-node bifurcation (SNp, at y≃0.1493) and drifts in the direction of decreasing *y* along a branch of attracting fixed points. The slow passage takes the trajectory through the Hopf bifurcation (H, at y≃0.0973) and eventually to the lower (stable) branch of fixed points. It then drifts in the direction of increasing *y* and leaves the branch of fixed points at the SNIC bifurcation (which coincides with the saddle-node of fixed points SNf at y≃0.0754). Finally, the trajectory is captured by the attracting branch of periodic orbits, which corresponds to the initiation of the active phase of the burst. Because the active phase of the burst initiates at the SNIC and terminates at the saddle-node of periodic orbits, this is a circle/fold cycle burster in the classification scheme of [7].

### 4.3 Torus canard explosion

The transition near the torus bifurcation at k≃−0.03852 in Figure 8 from rapid spiking to bursting occurs by way of a torus canard explosion. For values of *k* below the torus bifurcation, the periodic orbit of the full system (i.e., the rapid spiking state) is stable. As *k* increases above the torus bifurcation, the system exhibits AM spiking as the trajectory winds around the torus near the saddle-node of periodic orbits of the fast system. The torus grows as *k* increases, and parts of the torus shadow the attracting and repelling branches of periodic orbits of the fast system in alternation. Further increases in *k* lead the system through the torus canard explosion. This explosion consists of a sequence of distinct dynamics beginning with a rapid increase in amplitude of AM spiking corresponding to the headless ducks (Figure 8b), then MMO (Figure 8c), ducks with heads, and finally the complete circle/fold cycle bursters (Figure 9). Therefore, the torus canards play a central role in the transition from spiking to circle/fold cycle bursting in this model, just as was the case for the HR model in the transition to sub-Hopf/fold cycle bursting.

Figure 8c shows the time series of a trajectory at a value of *k* during the torus canard explosion where the system exhibits MMO dynamics. Each time the trajectory passes through the saddle-node of periodic orbits it transitions from the branch of attracting to the branch of repelling periodic orbits of the fast system, but the direction in which the trajectory leaves the repelling branch of periodic orbits varies from one pass to the next. When it falls outward toward the attracting branch of periodic orbits, it resembles the AM spiking and headless torus canard behavior seen at slightly smaller *k* values. When it falls inward toward the branch of fixed points, the trajectory resembles the bursting and torus canard with head trajectories seen at slightly larger *k* values.

### 4.4 Two-parameter bifurcation diagram and relation to other types of bursting

**Fig. 10:**
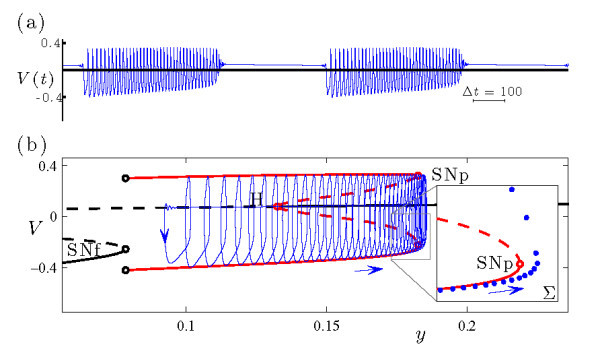
An example of sub-Hopf/fold cycle bursting in the MLT system (Equations 6a-6c) at $\left(k,g_{\mathrm{Ca}})=(-0.01,1.18\right)$. **(a)** Time series of the fast *V* variable. **(b)** The bursting trajectory (blue curve) is plotted in projection onto the (y,V) phase space, along with the bifurcation diagram of the fast system at this value of $g_{\mathrm{Ca}}$. Plotting conventions follow Figure 9.

In addition to the circle/fold cycle bursting described above, the MLT system also exhibits sub-Hopf/fold cycle bursting similar to that observed in the HR model in Section 3. An example of sub-Hopf/fold cycle bursting in the MLT system is shown in Figure 10, at $g_{\mathrm{Ca}}=1.18$. At this value of $g_{\mathrm{Ca}}$, the Hopf bifurcation H is located farther in *y* from the saddle-node of fixed points SNf that is associated with the SNIC (compare to Figure 9). In this case, the slow passage through the Hopf bifurcation does not take the trajectory to sufficiently small *y* to involve the SNIC. Instead, the bursting initiates when the trajectory spirals away from the unstable fixed point directly toward the attracting branch of periodic orbits of the fast system, creating a sub-Hopf/fold cycle burster. We note however that the transition (as the primary bifurcation parameter *k* increases) from spiking to the sub-Hopf/fold cycle bursting in Figure 10 goes by way of a torus canards explosion, just as it did for the circle/fold cycle bursting in Figure 9. In both cases, the torus canard explosion is associated with the dynamics near SNp. 

**Fig. 11:**
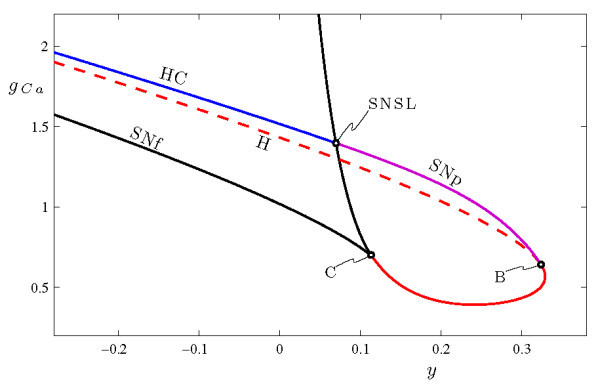
Two parameter bifurcation diagram of the fast system of Equations 6a-6c. The loci of saddle-nodes of fixed points SNf merge in a cusp bifurcation C at $\left(y,g_{\mathrm{Ca}})≃(0.1133,0.7016\right)$, which creates the locus of Hopf bifurcations H. The saddle-node of periodic orbits SNp emerge from the Bautin bifurcation B at $\left(y,g_{\mathrm{Ca}})≃(0.3238,0.6418\right)$ and extend to the saddle-node separatrix loop bifurcation SNSL at $\left(y,g_{\mathrm{Ca}})≃(0.06972,1.397\right)$, which also creates a locus of homoclinic bifurcations HC. There is a SNIC bifurcation on the segment of SNf between C and SNSL. Plotting conventions follow Figure 6.

 The common features in the bifurcation diagrams of the fast systems in Figures 9 and 10 suggest a close relationship between circle/fold cycle bursting and sub-Hopf/fold cycle bursting in the MLT model. Figure 11 shows how the various codimension-1 bifurcations from Figures 9 and 10 change as the secondary bifurcation parameter $g_{\mathrm{Ca}}$ varies. At large $g_{\mathrm{Ca}}$, the saddle-node of periodic orbits SNp disappears when it collides with the saddle-node of fixed points SNf associated with the SNIC in a codimension-2 bifurcation which is of saddle-node separatrix loop type, similar to what is studied in [40]; see also [41]. Above this value of $g_{\mathrm{Ca}}$, the branch of periodic orbits terminates in a homoclinic bifurcation HC involving the saddle fixed point. At smaller $g_{\mathrm{Ca}}$, the two saddle-nodes of fixed points collide in a cusp bifurcation C, which generates a second, supercritical Hopf bifurcation. Below the cusp, the SNIC is no longer possible and the branch of periodic orbits terminates instead in the newly formed Hopf bifurcation. There is also a codimension-2 Bautin bifurcation B as the original Hopf bifurcation changes from subcritical to supercritical, and the saddle-node of periodic orbits SNp terminates at this point. Thus, the MLT system includes a saddle-node of periodic orbits over a wide range of $g_{\mathrm{Ca}}$ values ($0.6418\leq g_{\mathrm{Ca}}\leq 1.397$). Circle/fold cycle bursting occurs at the higher end of this range, and sub-Hopf/fold cycle bursting occurs at the lower end. 

In summary, the MLT system exhibits different types of bursting behavior depending on $g_{\mathrm{Ca}}$. There is a wide range of $g_{\mathrm{Ca}}$ values for which the system exhibits some type of bursting involving a fold of limit cycles - either circle/fold cycle bursting or sub-Hopf/fold cycle bursting. In each case, the regimes of rapid spiking and bursting are separated by a torus canard explosion.

## 5 Torus canards in the Wilson-Cowan-Izhikevich system

 In this section, we consider the following extended version of the Wilson-Cowan model [42] proposed by Izhikevich in [7], which we call the Wilson-Cowan-Izhikevich (WCI) system:

(9a) $$\dot{x}=-x+S(r_{x}+ax-by+u),$$

(9b) $$\dot{y}=-y+S(r_{y}+cx-dy+fu),$$

(9c) $$\dot{u}=\varepsilon (k-x),$$

where S(x)=1/(1+exp(−x)). With ε≪1 the variables *x* and *y* are fast and *u* is slow.

As with the models considered in the previous sections, the WCI model can exhibit a wide variety of bursting dynamics. Here we are interested in this model as an example of a fold/fold cycle burster, where the active phase of the burst initiates in a fold of fixed points and terminates in a fold of limit cycles. Consistent with the results in the previous two sections, we first show that system (Equations 9a-9c) includes a torus bifurcation in the transition from spiking to bursting (Section 5.1) and that the fast system has a fold of limit cycles (Section 5.2), then describe the associated torus canard explosion that occurs during this transition (Section 5.3).

We treat *k* and $r_{x}$ as the primary and secondary control parameters, respectively, and fix

(10) $$\begin{array}{rl} r_{y} & =-9.7,\;a=10.5,\;b=10,\;c=10,\\ d & =-2,\;f=0.3,\;\varepsilon =0.03, \end{array}$$

for the remaining parameters.

### 5.1 Dynamics of the full system, and a torus bifurcation

**Fig. 12:**
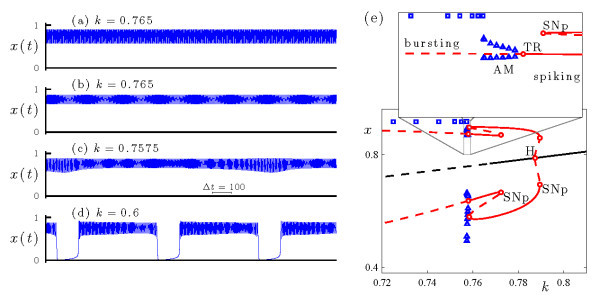
Dynamics of the WCI system (Equations 9a-9c) at $r_{x}=-4.76$. **(a)-(d)** Time series of the voltage variable *x* at several values of *k*, with *k* decreasing from top to bottom. The traces in frames (a) and (b) are at the same value of *k* and illustrate bistability. **(e)** Bifurcation diagram upon variations of parameter *k*, including branches of fixed points (black curve) and periodic orbits (two red curves, indicating maximal and minimal values of *x* over the orbit). Solid/dashed curves indicate stable/unstable solutions. The torus bifurcation at k≃0.7580 is supercritical. At smaller values of *k*, direct numerical simulations show that the system exhibits bursting (□, indicating maximal and minimal values of *x*) and AM spiking (△, indicating extrema of the modulation envelope).

Figure 12 summarizes the dynamics of the WCI model (Equations 9a-9c) at $r_{x}=-4.76$ during the transition from spiking to bursting. Frames (a)-(d) show the time series of the voltage variable *x* at several values of *k*, with *k* decreasing from top to bottom. At k=0.765, the system is bistable - it includes two stable spiking states with different amplitudes and frequencies (Figure 12a, b). At k=0.7575 the system exhibits AM spiking (Figure 12c), and at k=0.6 it exhibits bursting of fold/fold cycle type (Figure 12d). 

The bifurcation diagram of the WCI model (Equations 9a-9c) is presented in Figure 12e. It shows that this system has a branch of fixed points which loses stability as *k* decreases in a subcritical Hopf bifurcation (H, at k≃0.7874). The branch of periodic orbits that emerges from this Hopf point is unstable at onset, and its stability changes three times in three saddle-node bifurcations. This is the origin of the bistability of spiking states noted above. Finally, the branch destabilizes via a torus bifurcation (TR, at k≃0.7580). This torus bifurcation lies between the regimes of spiking and bursting dynamics, and is associated with torus canards.

### 5.2 Bifurcation analysis of the fast system

**Fig. 13:**
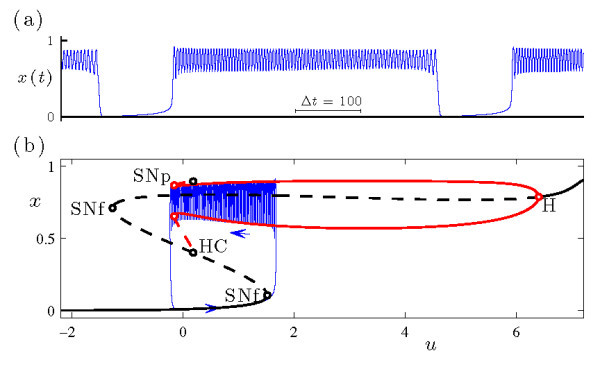
An example of fold/fold cycle bursting in the WCI model (Equations 9a-9c) at $\left(k,r_{x})=(0.6,-4.76\right)$. **(a)** Time series of the fast *V* variable. **(b)** The bursting trajectory (blue curve) is plotted in projection onto the (u,x) phase space, along with the bifurcation diagram of the fast system at this value of $r_{x}$. Plotting conventions follow Figure 2.

Figure 13 shows the bursting dynamics of the WCI model at k=0.6 and $r_{x}=-4.76$, previously shown in Figure 12d. The *x* time series in Figure 13a shows the two phases of the burst: the active phase of spiking and the inactive phase of quiescence. The *u* variable slowly decreases during the active phase and slowly increases during the inactive phase (not shown). 

Figure 13b shows the bursting trajectory plotted in projection onto the (u,x) phase space, which identifies this as a fold/fold cycle burster. The active phase of the burst initiates when the trajectory drifts in the direction of increasing *u* and falls off the branch of fixed points at a saddle-node of fixed points (SNf, at u≃1.517). During the active phase, the rapid spiking shadows the branch of stable periodic orbits of the fast system, and the slow variable *u* decreases. The active phase terminates when the trajectory drifts down and off the branch of periodic orbits at the saddle-node of periodic orbits (SNp, at u≃−0.1545), and returns to the stable branch of fixed points to repeat the cycle.

### 5.3 Torus canard explosion

**Fig. 14:**
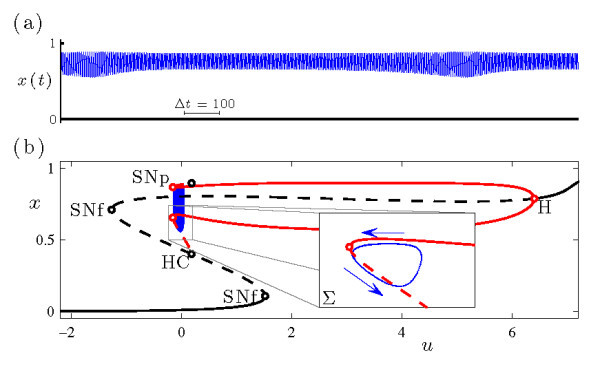
An example of a headless torus canard orbit in the WCI model (Equations 9a-9c) at $\left(k,r_{x})=(0.7575,-4.76\right)$. **(a)** Time series of the fast *V* variable. **(b)** The trajectory is plotted in projection onto the (u,x) phase space, along with the bifurcation diagram of the fast system at this value of $r_{x}$. Plotting conventions follow Figure 13. The inset shows the Poincaré map of the torus canard trajectory near SNp, with the Poincaré surface Σ chosen so that the iterates correspond to local extrema in *x* of the trajectory.

The transition from rapid spiking to bursting as *k* decreases through the torus bifurcation in Figure 12 occurs via torus canards. At the torus bifurcation point, the trajectory resembles the periodic orbit at the saddle-node of periodic orbits (SNp) of the fast system. At a value of *k* slightly below the torus bifurcation the trajectory winds around a torus near SNp, spending time, in alternation, near the attracting and repelling branches of periodic orbits of the fast system (see the headless torus canard trajectory shown in Figure 14). Further decrease of *k* completes the torus canard explosion (including MMO and ‘duck with head’ trajectories, not shown) and leads to the fold/fold cycle bursting trajectory shown in Figure 13. Moreover, the behavior at $r_{x}=-4.76$ is representative of a range of $r_{x}$ values in which the key ingredients for torus canards persists, and the WCI model (Equations 9a-9c) includes a transition from spiking to fold/fold cycle bursting via a torus canard explosion. 

### 5.4 Two-parameter bifurcation diagram and relation to other types of bursting

**Fig. 15:**
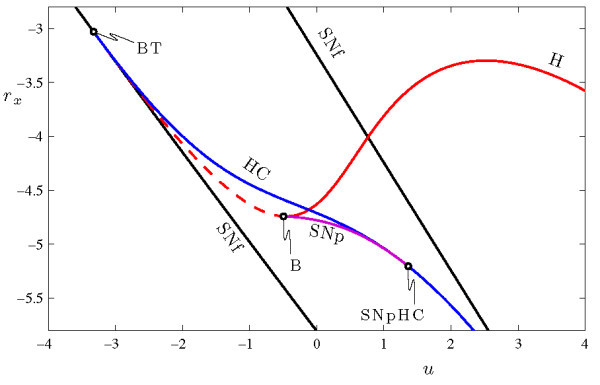
Two-parameter bifurcation diagram of the fast system of Equations 9a-9c in the $\left(u,r_{x}\right)$-plane. This includes loci of saddle-nodes of fixed points SNf, Hopf bifurcations H, saddle-nodes of periodic orbits SNp, and homoclinic bifurcations HC. There are three labeled codimension-2 bifurcations: a Bogdanov-Takens bifurcation BT at $\left(u,r_{x})≃(-3.325,-3.029\right)$, a Bautin bifurcation B at $\left(u,r_{x})≃(-0.4945,-4.740\right)$, and a SNpHC at $\left(u,r_{x})≃(1.364,-5.203\right)$. Plotting conventions follow Figure 6.

Figure 15 shows how the bifurcation structure of the fast system changes with the secondary control parameter $r_{x}$. Decreasing $r_{x}$ from $r_{x}=-4.76$ causes the saddle-node of periodic orbits SNp to disappear when it collides with the homoclinic bifurcation HC; this occurs at the codimension-2 point labeled SNpHC, at $r_{x}≃-5.203$. Increasing $r_{x}$ from $r_{x}=-4.76$ also causes the saddle-node of periodic orbits to disappear, but by a different mechanism. Increasing $r_{x}$ decreases the amplitude of the periodic orbits near SNp, and at sufficiently large $r_{x}$ ($r_{x}≃-4.741$), this amplitude shrinks to zero and the branch of periodic orbits collides with the upper branch of fixed points. This creates two new Hopf bifurcations by splitting the branch of periodic orbits into two pieces, one that connects the original Hopf bifurcation to one of the newly-formed Hopf points, and a second that connects the other newly formed Hopf to the homoclinic orbit HC. The latter branch includes SNp, but a codimension-2 Bautin bifurcation eliminates SNp at a slightly larger value of $r_{x}$ ($r_{x}≃-4.740$). Thus the saddle-node of periodic orbits persists over the range $-5.203<r_{x}<-4.740$. Further increase of $r_{x}$ eliminates one branch of periodic orbits as the supercritical Hopf bifurcations coalesce. There is also a codimension-2 Bogdanov-Takens bifurcation BT in which the subcritical Hopf and the homoclinic HC disappear. 

For values of $r_{x}$ above the Bautin bifurcation at $r_{x}=-4.74$, the fast system no longer includes a saddle-node of periodic orbits so bursters involving a ‘fold cycle’ are no longer possible. In this regime, the fast system includes a subcritical Hopf bifurcation (see Figure 15), and this can lead to new bursting scenarios. For example, it is possible to have a bursting orbit that follows the branch of attracting fixed points of the fast system down in *u* to the subcritical Hopf bifurcation and then spirals along the associated branch of repelling periodic orbits for some time.

The saddle-node of periodic orbits SNp persists as $r_{x}$ decreases down to the SNpHC point. Below this point the active phase of the bursting cycles terminates at the homoclinic orbit (i.e., fold/homoclinic bursting). We note however that the torus bifurcation of the full system only persists down to $r_{x}≃-5.10$. Below this value the stable periodic orbits of the full system lose stability in a period doubling bifurcation instead, so the transition from spiking to bursting does not involve torus canards.

## 6 Conclusions and discussion

### 6.1 Summary

 Torus canards were originally identified in a fifth order model of a Purkinje cell [21], where it was shown that the torus canard explosion occurs within the transition region between tonic spiking and bursting. Some basic aspects of the dynamics of torus canards were studied in [20] in the context of an elementary third order model, obtained by rotating a planar bistable system of van der Pol type and introducing symmetry-breaking terms. In this article, we extended this work and presented two primary results. First, we showed that torus canards are common among model neuronal systems of fast-slow type for which the fast systems have a saddle-node of periodic orbits (a.k.a. a fold of limit cycles) and the full systems have a torus bifurcation. The torus canard orbits spend long times near branches of attracting and repelling periodic orbits of the fast system in alternation, switching over from the former to the latter exactly near the saddle-node of periodic orbits. Moreover, these torus canards are the natural analog in one higher dimension of the by-now classical canards of limit cycle type, which spend long times near branches of attracting and repelling fixed points in alternation, as for example in the van der Pol and FitzHugh-Nagumo equations [28,43]. It was shown here that the Hindmarsh-Rose (HR) system, the Morris-Lecar-Terman (MLT) model, and the Wilson-Cowan-Izhikevich (WCI) model all have the essential ingredients to possess torus canards, namely a saddle-node of periodic orbits in the fast system and a torus bifurcation in the full system. Also, we described in detail the families of torus canards that exist in these models, and identified the torus canard explosions.

Second, we demonstrated that the torus canard explosions in these systems play central roles in the transitions between the spiking and bursting regimes. In the HR system, the torus canards occur precisely in the transition region from spiking to sub-Hopf/fold cycle bursting, in which the active phase of the burst initiates when the trajectory passes a subcritical Hopf bifurcation point and terminates when it passes the fold of limit cycles. The transitions from spiking to bursting in the MLT and WCI models are, respectively, to circle/fold cycle bursting in which the active phase initiates in a saddle-node bifurcation on an invariant circle (a.k.a. SNIC), and to fold/fold cycle bursting in which the active phase initiates as the trajectory passes a fold of fixed points.

### 6.2 On the topological necessity of torus canards

For the three neuronal models studied in this article, a topological argument may be given to show why torus canards must occur in the transition regime from rapid spiking to bursting. This topological argument complements the numerical and analytical results presented in this article, and it is analog to the topological argument that has been used to demonstrate the existence of classical limit cycle canards in planar systems such as the van der Pol and FitzHugh-Nagumo equations.

 From Section 2.1, we recall that, in such planar systems, the explosion of limit cycle canards occurs during the transition from equilibrium to periodic relaxation oscillations. The attracting set expands from being a point (zero-dimensional set) to being a limit cycle (closed curve) as soon as the Hopf bifurcation curve has been crossed. Moreover, as the parameter grows beyond the Hopf point, the amplitude of the limit cycles increases continuously from small to large through the sequence of limit cycle canards, first of the headless variety and then of the variety with heads, as shown in Figure 1, for example. The property of continuous dependence of solutions on parameters forces the deformation to pass continuously through this explosion of limit cycle canards in order to transition from equilibrium to the full-blown relaxation oscillations in these planar systems. There is no other way in the plane for this transition to occur in a continuous manner. This was the fundamental insight of earlier studies, see [28].

In the third-order neuronal models studied here, the rapid-spiking solutions - which exist for parameter values before the torus bifurcation values - deform continuously into bursting solutions as the parameter increases beyond the torus bifurcation point. This transformation must be continuous due to the continuous dependence of solutions on parameters. Moreover, as is the case for all orbits of a smooth ordinary differential equation, the solutions must be tangent to the vector field at all points along the orbits for each value of the parameter in this transition interval. Then, by examining how this transition can take place, we find that the only path, i.e., the only allowable homotopy from spiking to bursting, in these third-order systems is through the sequence of torus canards, both of the headless variety and with head, as observed herein. The periodic spiking solutions are one-dimensional attractors, and the bursting solutions wrap themselves tightly around a two-dimensional surface (near the manifolds made up of families of attracting and repelling limit cycles in the fast system) with a handle (the portion near the branch of slowly-varying equilibria in the fast system). The only way that the former can deform into the latter is by having solutions for intermediate parameter values that get stretched over the surface formed by the attracting and repelling periodic orbits.

Finally, on this topic, we observe that, while this topological argument establishes that solutions transition through the family of headless torus canards and then the torus canards with head (as has just been shown), it is insufficient to determine the monotonicity of this transition. Monotonicity is, as yet, only known based on numerical simulations. That determination requires analytical work, just as has been done for the monotonicity of the explosion of limit cycle canards in the van der Pol and FitzHugh-Nagumo equations. This topic is an important future study.

### 6.3 Outlook

 To conclude this article, we discuss other neuronal systems in which torus canards might occur. We propose that torus canards exist in other models that exhibit the types of bursting - sub-Hopf/fold cycle, circle/fold cycle, and fold/fold cycle - studied here. For example, the top-hat burster of Best et al. [44] is known to exhibit fold/fold cycle bursting and we therefore hypothesize that torus canards also appear in this model, although there may exist some technical differences since this is a fourth-order model.

 There are other classes of bursting dynamics in which the active phase of the burst terminates in a fold of limit cycles, but in which the initiation event is different from those considered here. For example, from the classification in Table 1.6 of [7], one sees that there are also super-Hopf/fold cycle bursters. For these, the active phase of the burst initiates with a supercritical Hopf bifurcation. However, since the termination event is also a fold of limit cycles, we hypothesize that these bursters will also exhibit torus canards. We note that, for these super-Hopf/fold cycle bursters, the slow passage effect through a Hopf bifurcation will play a role in determining the system parameters for which torus canards exist, just as it did for the sub-Hopf/fold cycle bursters.

 In addition, while we have only examined bursters in which the initiation event involves bifurcations of fixed points, there are also bursters in which the burst-phase is triggered by the bifurcation of an invariant set of dimension greater than zero, such as a limit cycle or torus. Also, we refer the reader to [45] for a natural catalog of the bifurcations that can initiate and terminate the active phase of bursting in fast-slow systems. There, low-codimension singularities in the fast systems are analyzed in a systematic fashion, and the slow variables are used as the unfolding parameters. The natural catalog is generated by identifying all possible paths that lead to bursting in these unfolding spaces. We think that, as long as the burst phase terminates in a fold of limit cycles, these systems may also exhibit torus canards, as well as new categories of canards that spend time near other types of attracting and repelling sets, not just limit cycles, and in various sequences [46].

 Finally, the question of whether or not tori in these neuronal models undergo breakdown due to resonances is a subject for future investigation. In general, one expects systems with Neimark-Sacker bifurcations to tori to exhibit resonances for certain parameter values, see for example [47,48]. This is in analogy to the formation of Arnold tongues in circle maps, for instance. In addition, the breakdown of tori due to resonances is known to lead to complicated chaotic dynamics.

## Appendix 1: Purkinje model

 In this appendix, we state the parameter values and forward and backward rate functions for the Purkinje cell model (Equations 3a-3e), taken from [49]. The parameters are given in Table 1, and the rate functions are: (11a)(11b)(11c)(11d)(11e)(11f)(11g)(11h)

 The equilibrium function for the fast sodium gating variable is

(12) m,NaF∞=[1+e−(V+34.5)/10]−1.

The sodium channel is sufficiently fast that we make the standard approximation in Equation 3a that  takes the value m,NaF∞. 

**Table 1:** Parameters used in the Purkinje cell model (Equations 3a-3e). In addition, we use C=1 nF for the cell’s capacitance.

Channel	Reversal potential (mV)	Conductance (*μ*mho)
Leak (L)		
High-threshold calcium (CaH)		
Fast sodium (NaF)		
Delayed rectifier potassium (KDR)		
M-current (KM)		

## Competing interests

The authors declare that they have no competing interests.

## Authors’ contributions

JB and MK conceived of the analysis and of the overall goals of the project. JB, MD, TK, and MK each suggested one of the bursting models included in the article. AB carried out analysis and numerical simulations for the Hindmarsh-Rose and Morris-Lecar-Terman models. JB and MD carried out additional calculations and numerical simulations for all three model bursters. MD developed the specialized continuation strategy which was used for this project. TK contributed the topological argument about the necessity of torus canards. All authors read and approved the final manuscript.
